# Pemphigoid disease model systems for clinical translation

**DOI:** 10.3389/fimmu.2025.1537428

**Published:** 2025-03-17

**Authors:** Marvin Tigges, Sören Dräger, Ilaria Piccini, Katja Bieber, Artem Vorobyev, Janin Edelkamp, Marta Bertolini, Ralf J. Ludwig

**Affiliations:** ^1^ QIMA Life Sciences, QIMA Monasterium GmbH, Münster, Germany; ^2^ Department of Dermatology, University Medical Center of the State of Schleswig-Holstein (UKSH), Lübeck, Germany; ^3^ Lübeck Institute of Experimental Dermatology, University of Lübeck, Lübeck, Germany

**Keywords:** pemphigoid diseases, model systems, pathogenesis, treatment, bullous pemphigoid, mucous membrane pemphigoid

## Abstract

Pemphigoid diseases constitute a group of organ-specific autoimmune diseases characterized and caused by autoantibodies targeting autoantigens expressed in the skin and mucous membranes. Current therapeutic options are still based on unspecific immunosuppression that is associated with severe adverse events. Biologics, targeting the IL4-pathway or IgE are expected to change the treatment landscape of pemphigoid diseases. However, clinical studies demonstrated that targeting these pathways alone is most likely not sufficient to meet patient and healthcare partitioners expectations. Hence, model systems are needed to identify and validate novel therapeutic targets in pemphigoid diseases. These include pre-clinical animal models, *in vitro* and *ex vivo* model systems, hypothesis-driven drug repurposing, as well as exploitation of real-world-data. In this review, we will highlight the medical need for pemphigoid diseases, and in-depth discuss the advantages and disadvantages of the available pemphigoid disease model systems. Ultimately, we discuss how rapid translation can be achieved for the benefit of the patients.

## Introduction

**Figure 1:**
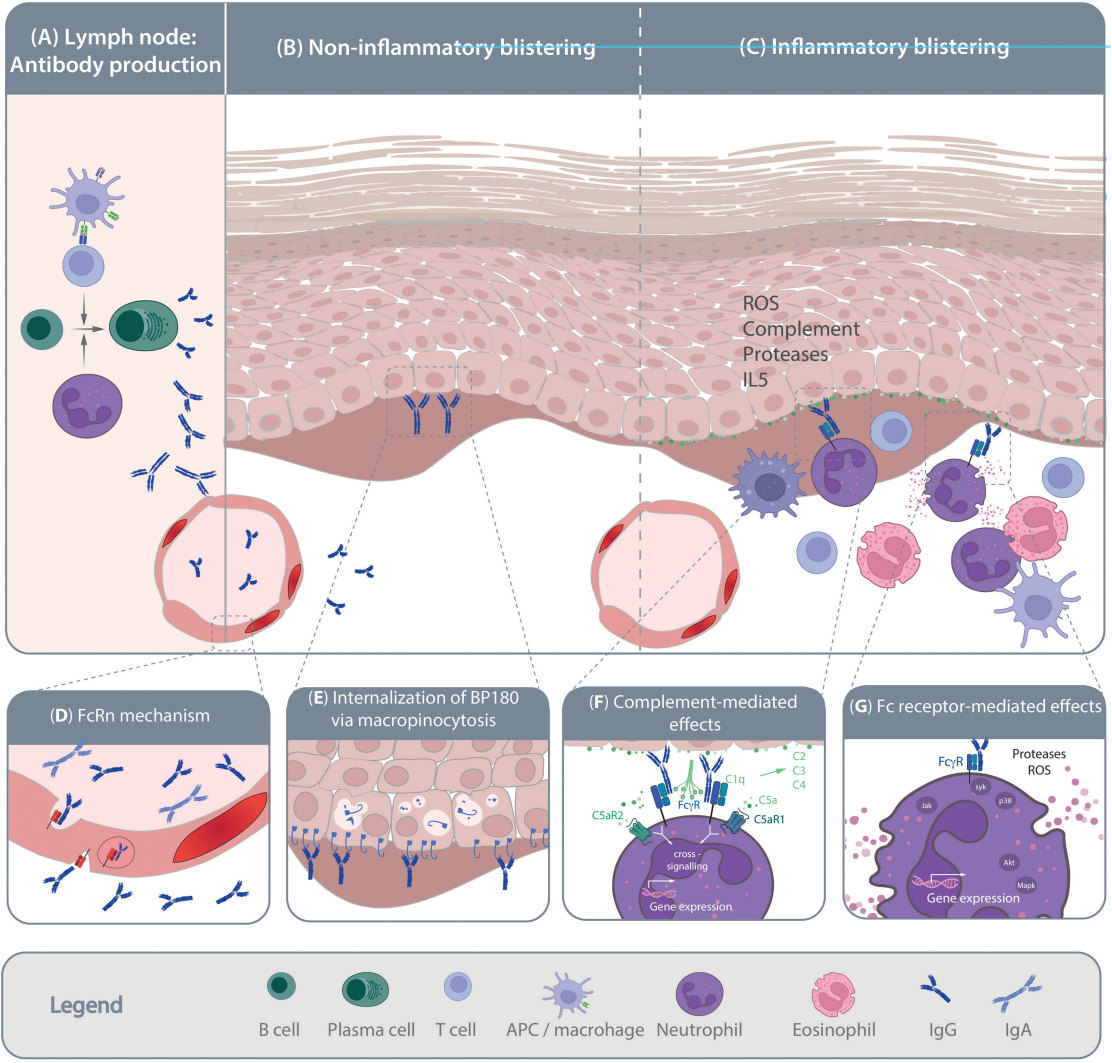
Pathogenesis of pemphigoid diseases. Schematic of the current understanding of pemphigoid disease pathogenesis. **(A)** Loss of tolerance leads to the generation of autoantigen-specific B cells. Activation, proliferation and maturation of B cells occurs in a T cell-depended manner and is further promoted by the presence of neutrophils. Mechanisms leading to the shift from the production of non-pathogenic towards pathogenic autoantibodies are so far poorly understood. Pathogenic autoantibodies are then released into the circulation and reach the skin through the vasculature. Autoantibody binding to the target antigens in the skin and/or mucous membranes can trigger blister formation through **(B)** non-inflammatory and/or **(C)** inflammatory mechanisms. Non-inflammatory blistering antibody binding to the target antigen, specifically BP180, induces the internalization of BP180, leading to destabilization of keratinocyte adherence to the basement-membrane. Inflammatory mechanism leading to blister formation in pemphigoid disease is relatively well-understood. These include release of pro-inflammatory mediators from the targeted cells and immune cells, which facilitates myeloid cell migration into the skin. Within the skin, myeloid cells bind to the tissue bound immune complexes and mediate blistering trough protease- and reactive oxygen species (ROS) release. The inserts **(D-H)** provide some more details on the highlighted pathogenic events. **(D)** Within the circulation the half-life of IgG autoantibodies is prolonged by the neonatal Fc receptor (FcRn). **(E)** After binding of anti-BP180 antibodies, BP180 is internalized through micropinocytosis. **(F)** Complement, especially C5 is release from both keratinocytes and myeloid cells. Cleavage of C5 into C5a recruits and activates myeloid cells to the site of autoantibody deposits in the skin that is mediated through both C5aR1 and C5aR2. **(G)** Ultimately, myeloid cells engage the tissue-bound immune complexes in a Fc gamma receptor-mediated fashion, leading to protease- and ROS-release. These processes are mediated by downstream intracellular signaling following Fc gamma receptor engagement.

Pemphigoid diseases (PD) constitute a group of rare autoimmune skin disorders. Based on target antigen, autoantibody isotype and clinical presentation, seven PD subtypes are differentiated (1). Of note, despite recent advances (2) there are still unrecognized autoantigens in a minority of PD patients (3, 4). PD are characterized by autoantibodies targeting components of the dermal-epidermal junction, resulting in split formation and inflammation. This process typically necessitates the activation of myeloid cells, but events triggered by target engagement of autoantibodies have also been directly linked to subepidermal blister formation (**Figure 1**). The subepidermal split formation is characteristic for PD and differentiates them from other autoimmune bullous dermatoses (AIBD) (5). In this section, we provide a brief introduction into each PD and highlight the specific unmet medical need.

## Bullous pemphigoid

Bullous pemphigoid (BP) is the most common pemphigoid disease, with an incidence of 20 cases per million in Germany (6). However, this incidence varies globally and is increasing over time (7). A significant factor contributing to this rise is the aging population and the use of medications that can trigger BP (8). BP predominantly manifests in individuals in their late 70s. In those aged over 80 years, the incidence surges to 150-330 cases per million per year (1, 9–14). The clinical presentation of BP is heterogenous, ranging from pruritus without evidence of skin lesions to dense blistering on inflamed skin (15) and is characterized by the autoimmune response targeting two structural components of hemidesmosomes, namely BP180 (type XVII collagen, COL17) and BP230. These proteins serve to link the cytoskeleton of basal keratinocytes to structures in the papillary dermis (16). Antibodies are usually of the immunoglobulin (Ig)G subclass, but IgA- and other subclasses have also been documented to cause BP (16). Autoantibodies in PD develop in a T-cell dependent B cell response (17). As outlined in **Figure 1**, binding of autoantibodies to their target epitopes initiates pathology through inflammation- dependent and -independent mechanisms (18, 19). Diagnosis of BP is based on clinical presentation and histopathological analysis. The diagnosis is confirmed by detection of Ig or complement C3 deposition at the dermal-epidermal junction using direct immunofluorescence (DIF), and the detection of circulating BP180 and/or BP230 autoantibodies (20). Treatment of BP centers on topical or systemic corticosteroid treatment and/or systemic immunosuppression. Corticosteroid treatment is highly effective with over 90% achieved remissions within 4-6 weeks (21–25). The major challenge in BP is maintenance of this initial very effective treatment response. Depending on diseases severity, 30%-50% of the patients experience a relapse within 6 months after the initial diagnosis (26). This, in turn, necessitates prolonged corticosteroid treatment, which contributes to the high morbidity and increased mortality in BP (27, 28).

## Mucous membrane pemphigoid

Mucous membrane pemphigoid (MMP) is defined as a PD with predominant mucosal involvement. Studies estimate its incidence at 2.0 per 1 million people per year in the state of Franconia (Germany) with a prevalence of 24.6 per million people in Germany (1, 11, 29, 30). The disease typically manifests at a mean age of 60 to 65 years (31). MMP primarily affects mucous membranes in the mouth, nose, eyes, anogenital area, pharynx, larynx, and esophagus, but can also involve the skin. The most common affected site is the oral mucosa, followed by eyes and nose (32). Severity varies highly among patients and has a range from subtle lesions to devastating esophageal and conjunctival lesions that are extremely painful and may lead to esophageal strictures and blindness (31, 33, 34). MMP can be caused by autoantibodies targeting several different antigens, including BP180, BP230, laminin 332, COL7, and/or integrin α6β4. Among these, autoantibodies against BP180 are the most common (1, 32, 35, 36). If clinically suspected, MMP diagnosis is confirmed by the detection of immunoglobulin and/or C3 deposits using DIF (1). Of note, in cases where clinical suspicion of MMP is high, but DIF is negative, repeat biopsies should be conducted to increase the sensitivity of DIF microscopy (37). Although sometimes challenging, detecting circulating autoantibodies and determining their specificity should be attempted (30). MMP is among the more challenging pemphigoid diseases to treat, often requiring a combination of immunosuppressants to achieve remission. In MMP with autoantibodies against laminin 332, a 25-30% prevalence of mainly solid tumors has been described (38). The prognosis of MMP varies depending on the organs affected. Overall, mortality in MMP patients is increased 1.7-fold compared to matched controls (28).

## Epidermolysis bullosa acquisita

Epidermolysis bullosa acquisita (EBA) is an orphan disease with an annual incidence of 0.2.-0.5 new cases per million people (11, 39, 40). EBA can occur at any age, with a mean age at onset of 46.7 years (29, 41–43). Like BP, the clinical presentations varies greatly in EBA patients. The two most common forms are the inflammatory (or non-mechano-bullous) and the mechano-bullous form. The mechano-bullous EBA presents with skin fragility, blisters and erosions on non-inflamed skin or scarred skin and millia formation. These symptoms predominantly occur on trauma-prone areas such as the hands, feet, elbows, and knees (18). Inflammatory EBA presents with profuse skin lesions on inflamed skin, similar to BP, and trauma induced lesions around non-inflamed skin (18, 44–46). Mucosal involvement is common in EBA (34, 47). EBA is caused by IgG or IgA autoantibodies against type VII collagen (COL7), commonly targeting the immunodominant NC1 region (48). In approximately 1/3^rd^ of EBA patients IgA autoantibodies are detected, and in 10% of the patients this is the sole detected immunoglobulin subclass. Similar to BP, binding of these autoantibodies to their target antigen initiates an inflammatory cascade resulting blistering and inflammation. Mechanisms of non-inflammatory blistering in EBA are less well characterized (18). EBA diagnosis is confirmed by linear Ig and/or complement C3 deposits the dermal-epidermal junction of perilesional skin in DIF. Contrasting all other pemphigoid diseases, Ig deposition at the dermal-epidermal junction shows a distinct, so-called u-serrated binding pattern while all other PDs display an n-serrated pattern in DIF microscopy. Indirect IF microscopy on human salt-split skin or ELISA further supports the diagnosis by detecting COL7 autoantibodies or its immunodominant domains (1, 48, 49), but should only be used in addition of serration analysis because in a large proportion of EBA patients circulating autoantibodies cannot be detected (50, 51). If serration analysis is not possible, alternatives are fluorescent overlay antigen mapping or immunoelectron microscopy (18). The treatment of EBA is notably difficult. Typically, achieving remission necessitates extensive immunosuppression (43). On average, a continuous immunosuppressive treatment regimen of nine months is required to achieve remission (52). If remission is achieved, in many cases, continued immunosuppression is needed to maintain the therapeutic effects. Data on mortality in EBA are scant. However, a recent retrospective cohort study found that EBA patients have an approximate 2.5-fold increased risk of mortality compared to matched controls (28).

## p200 pemphigoid

Anti-p200 pemphigoid is a rare autoimmune blistering disease characterized by subepidermal blisters and erosions primarily on the skin and sometimes on mucosal surfaces. The disease is defined by autoantibodies targeting the 200-kDa protein lamininβ4, a key component of the basement membrane zone (2). Clinically, anti-p200 pemphigoid presents with tense blisters, erythematous plaques, and urticarial lesions, predominantly affecting the hands and feet (53). Diagnosis involves detecting linear IgG deposits along the basement membrane zone via DIF and identifying circulating autoantibodies against laminin β4 (53). The presence of these autoantibodies is critical for differentiating anti-p200 pemphigoid from other similar blistering diseases. Treatment typically includes topical or systemic corticosteroids and immunosuppressive agents to manage inflammation and autoantibody production. The prognosis for patients with anti-p200 pemphigoid varies, with early and aggressive treatment potentially improving outcomes and reducing the risk of complications (53, 54).

## Pemphigoid gestationis

Pemphigoid gestationis (PG) is a pregnancy-caused PD with an estimated incidence of 1 in 20,000 to 50,000 pregnancies per year (1, 55, 56). Onset of the disease is typically between the second trimester and the postpartum period (57). PG manifests with pruritus and polymorphic inflammatory skin lesions spreading from the umbilical region to the abdomen and extremities. In most cases, PG resolves after delivery. However, relapses during subsequent pregnancies are common (1, 57). PG is caused by autoantibodies targeting BP180. Similar to other PD, PG is diagnosed by the detection of tissue-bound immunoglobulins and/or complement C3 in DIF. Circulating BP180 antibodies can be detected using indirect IF with salt-split skin as substrate or with specific ELISAs. Topical corticosteroids can treat most cases of PG effectively. Interdisciplinary care involving gynecology is recommended due to the increased risk of adverse embryonic/fetal and maternal pregnancy outcomes associated with PG (58–60). Of note, approximately 10% of newborns exhibit PG-typical skin lesions due to the transplacental transfer of maternal IgG antibodies (61, 62), which may require supportive neonatal care.

## Linear IgA disease

Linear IgA disease (LAD) manifests across all age groups, with the highest incidence observed during adolescence, early adulthood, and the sixth decade of life. In pediatric populations, it is the most prevalent autoimmune blistering disorder, with an average onset age of 4.5 years. The annual incidence is estimated to range from 0.2 to 2.3 cases per million individuals (63). Clinically, LAD presents with tense blisters and vesicles, often forming in a characteristic “crown of jewels” or “string of pearls” pattern, particularly in children. Lesions can appear on both the skin and mucous membranes, causing significant discomfort due to associated pruritus (1, 64). LAD is caused by IgA autoantibodies targeting the extracellular 97 kDa portion of BP180 and the 120 kDa ectodomain of BP180, known as LAD-1 (63, 65, 66). Diagnosis of LAD is primarily based on DIF microscopy, which shows linear IgA deposits along the basement membrane zone. Identification of circulating antibodies targeting LAD-1 supports the diagnosis (67). Dapsone is the treatment of choice for LAD, which, in most cases rapidly alleviates itch, inflammation and blister formation. In children, the disease often resolves spontaneously within a few years. In adults, however, LAD may follow a more chronic course, requiring prolonged treatment (68, 69).

## Lichen planus pemphigoides

Lichen planus pemphigoides (LPP) is an orphan AIBD that combines features of lichen planus (70) and bullous pemphigoid. It is characterized by the presence of lichenoid papules and plaques, which subsequently develop tense blisters. LPP is at least partially caused by autoantibodies targeting BP180. Clinically, LPP presents with intensely pruritic, violaceous, polygonal papules and plaques that are often located on the extremities. The blisters usually develop on skin areas that were previously unaffected by lichen planus lesions. Diagnosis is confirmed through DIF, which shows linear deposits of IgG and/or C3 at the basement membrane zone, and the presence of circulating autoantibodies targeting BP180. The pathogenesis of LPP is incompletely understood. It potentially involves an initial lichenoid inflammation that may promote an autoimmune response against components of the epidermal basement membrane, particularly BP180. This could lead to the formation of BP180-specific autoantibodies that ultimately lead to subepidermal blistering. Treatment options for LPP include systemic corticosteroids, which are often the first line of treatment. LPP usually responds well to treatment, but more definite data on LPP prognosis is scant due to the rarity of the disease (71).

## Current treatment and unmet medical need in pemphigoid diseases

PD impose a significant morbidity and dramatically impair the quality-of-life of the affected patients. Furthermore, PD patients bear an increased mortality risk, e.g., a 1-year mortality ranging from 20% to 40% in BP (13, 14, 28, 72). While PG, LAD, and p200 pemphigoid generally respond favorably to therapeutic interventions, BP, MMP and EBA are notably more challenging to manage (73). These latter PD often relapse and typically require prolonged courses of immunosuppressive therapy to achieve remission (1, 73). Initial treatment of PDs mostly consists of systemically or (in BP) topically applied corticosteroids (27). The administration of systemic corticosteroids, such as prednisone, in elderly patients links to significant adverse effects and an increased risk of mortality (21, 22, 74). Potent topical corticosteroids, such as clobetasol propionate, exhibit fewer side effects compared to systemic corticosteroids. However, their use is limited by corticosteroid-induced skin atrophy and practical challenges, particularly in elderly patients (21, 75). Long term treatment is usually centered on corticosteroid sparing immunosuppressive drugs (73).

Second- and third-line treatments for PD include immunosuppressants, high doses of intravenous immunoglobulins, rituximab, or more recently use of biologics such as dupilumab or omalizumab. For most PD treatment guidelines are available. For detailed insights we refer to those guidelines (23, 76–79). The use of biologics in PD is detailed below when describing drug repurposing to develop novel treatment options for PD.

The study from Lamberts and colleagues (80) revealed that patients, clinicians and researchers agreed that the most urgent need was improvement of therapeutic options for pemphigoid diseases. Furthermore, half of the patients were unsatisfied with patient care during the diagnostic process due to misdiagnosis and long diagnostic delay. PD patients also express the desire for increased disease awareness among healthcare professionals to facilitate more accurate and timely diagnosis. Researchers, on the other hand, seek an increase in clinical trials, a deeper understanding of disease pathophysiology to aid in drug development, and an exploration of trigger factors. Besides unmet patient needs, the increasing incidence of BP and limitations of currently available treatment options, e.g. incomplete response rates, severe side effects and costs underline the need for improvement in the aforementioned areas and the need to keep developing novel, more efficient and specific treatments. Use of pre-clinical models for target identification and -validation is one possibility to improve long-term therapeutic outcomes in PD.

## Pre-clinical PD model systems

Pre-clinical studies of PDs are mostly reliant on *in vivo* animal models. These models allow to investigate autoantibody-induced interactions of specific molecules underlying the pathomechanisms and in identifying suitable therapeutic target candidates (81–84). The following section summarizes the available pre-clinical animal models for PDs, their applications, their respective advantages and disadvantages. In principle, three different types of animal models are utilized in PD research (**Table 1**): (Auto)-antibody transfer models, lymphocyte-transfer models and immunization-induced models. Spontaneous PD, found primarily in dogs and occasionally in other domestic animals, is a rare occurrence and is only rarely used for drug development (83, 85). In addition, the deletion of the NC14A domain in mice leads to the spontaneous loss of tolerance and the mice subsequently develop BP-like symptoms (86).

**Table 1:** Pre-clinical models of pemphigoid disease.

Pemphigoid disease	Mode of induction	Species (most commonly used strains)	Disease Penetrance	Comments	Advantages	Disadvantages	Reference
Spontaneous pemphigoid diseases in animals							
BP	Spontaneous	Cat	rare	Very rare and time of disease outbreak is not controllable	• Closely resembling BP in humans	• Very rare and time of disease outbreak is not controllable	(197)
BP	Spontaneous	Dog	rare	–	–	–	(198) (199)
BP	Spontaneous	Pig	rare	–	–	–	(200)
BP	Spontaneous	Horse	rare	–	–	–	(201)
BP	Spontaneous	Mice	High	Deletion of the NC14A domain in the mice required	Closely resembling BP in humans	–	(86)
MMP	Spontaneous	Dog	rare	–	–	–	(202)
EBA	Spontaneous	Dog	rare	–	–	–	(203)
Antibody transfer-induced pemphigoid disease							
BP	Rabbit anti-mouse COL17	Neonatal mice (BALB/c)	100%		• High penetrance • Fast	• Use of neonatal mice • Pharmacological intervention challenging	(87)
BP	Rabbit anti-mouse COL17	Adult mice (SJL, BALB/c,C57BL/6J, C57BL/10)	100%		• High penetrance • Stable and extensive disease induction	• Pre-sensitization with rabbit IgG necessary	(88)
BP	Sheep anti-mouse COL17	Adult mice (BALB/c)	100%	Sheep IgG led to an earlier onset and more active disease compared to rabbit IgG	• High penetrance	• Later disease onset • Lesion development mostly limited to side of IgG injection	(204)
BP	Rabbit anti-mouse COL17	Adult mice (BALB/c, C57BL/6J)	100%	Disease inducing effect of IgG is dose-dependent	• High penetrance • Reproduce major clinical and immunopathological characteristics of human disease		(89)
BP	Mouse anti-mouse BP230	Neonatal scurfy mice	Disease development in the majority of mice		• Blistering over the whole body	• Use of neonatal mice • Only development of microscopic blisters • Pharmacological intervention challenging	(205)
BP	Mouse anti-mouse NC14-1	Adult mice (C57BL/6J)	100%		• High penetrance • Extensive blister development • Early lesion development	• No induction of mucosal lesions	(206)
BP	patient IgG	Neonatal mice (COL17 ^m−/–,h+^)	100%		• Use of human IgG • Fast erythema development • High penetrance • Close to human BP	• Use of neonatal mice • Pharmacological intervention challenging	(90)
BP	anti-human BP180NC16A	Neonatal mice (BP180NC16A(NC16A^+/+^))	100%		• High penetrance • Use of anti-human IgG • Quick lesion development	• Use of neonatal mice • Number of eosinophils in infiltrate does not reflect human BP • Pharmacological intervention challenging	(91)
BP	monoclonal anti-human COL17	Neonatal mice (COL17 ^m–/–,h+^)	88%		• Similar results to patient IgG usage	• Use of neonatal mice • Pharmacological intervention challenging	(207)
EBA	Rabbit or human anti-mouse COL7	Adult mice (C57BL/6J, BALB/c)	100%	Many susceptible strains with varying disease penetrance	• High penetrance	• Anti-rabbit (or human) IgG immune response	(208)
EBA	rabbit anti-COL7	Adult mice (SKH1)	100%	Affected body area is IgG dose-dependent	• High penetrance • Blistering at different regions of the body • Longer lasting disease phenotype	• No mucosal involvement often found in human EBA	(209)
EBA	affinity-purified human anti-CMP	Adult mice (SKH1)	100%		• High penetrance • Use of human IgG		(210)
EBA	affinity-purified rabbit anti-mouse COL7	Adult mice (BALB/c)	Not stated	Dependent on COL7-domain specificity, antibodies exhibit different disease phenotypes	• Development of extensive and widespread lesions		(211)
EBA	rabbit anti-mouse COL7	Adult mice (BALB/cJ, C57BL/6J)	100%	Dose-dependent onset and lesion development	• Used IgG exhibits cross-reactivity with human skin • High penetrance	• Skin blistering induction is strain-dependent	(212)
EBA	rabbit anti-mouse COL7	Adult mice (BALB/cJ, C57BL/6J)	Not stated	Investigated the involvement of T-cells on disease development			(213)
EBA	rabbit anti-human COL7	Adult mice (COL7 ^m−/−,h+^)	Not stated	Antibodies to different COL7 epitopes were tested. Dose-dependent effect on skin lesions	• Only available humanized EBA model		(214)
MMP	rabbit anti- LAM332	Neonatal mice (BALB/c, DBA/2NCr, W/W^v^)	100% at higher concentrations	Lesions developed predominately at sides of friction and trauma	• Quick development of microscopic blisters	• Use of neonatal mice • Dose-dependent disease severity • Pharmacological intervention challenging • No development of generalized disease observed	(215)
MMP	Rabbit anti-mouse LAM332	Adult mice (BALB/c, C57BL/6, a.o.)	Not stated		• Reflects major histo- and immunopathological characteristics of human MMP		(216)
MMP	rabbit anti- LAM332	Adult mice (BALB/c, DBA/2NCr, a.o)	100% except lowest concentration		• Quick occurrence of subepidermal blistering • Pharmacological interventions possible	• No development of frank blistering • Disease development strain-dependent	(217)
MMP	Patient IgG/rabbit anti- LAM332	Adult mice(SCID (human skin engrafted))	100% except lowest concentration		• Usage of human IgG possible	• Higher resistance of human skin to blistering • Higher antibody doses required	(217)
LAD	monoclonal mouse IgA and anti-human COL17	Adult mice(SCID (human skin engrafted))	High IgA disposition and split formation at BMZ but rare blister development		• Only LAD model available	• No full disease development • Low IgA titers used	(218)
Lymphocyte transfer-induced pemphigoid disease							
BP	immunized splenocytes from human skin graftedCOL17 ^m−/−,h+^	Adult mice (Rag-2−/−/COL17 ^m−/−,h+^)	70-80% of mice develop skin changes but 100% display histopathological markers		• Targeting of human immune response possible • Active and stable model	• Several steps required	(104)
Immunization-induced pemphigoid disease							
BP	Immunization	Mice (female SJL/J)	55% of female mice developed skin lesions but none of the male mice		• Stable disease model	• Limited to female mice	(108)
EBA	Immunization	Mice (SJL/J and B6.SJL-H2^s^ C3^c/^1CyJ)	45-100% development of skin blistering phenotype in susceptible strains	Repeated and single immunization possible	• Stable disease model	• Strain dependent disease development	(107) (109)
EBA	Immunization	Mice (SJL)	100%		• High penetrance • Stable disease model		(211)

BP, Bullous pemphigoid; MMP, Mucous membrane pemphigoid; EBA, Epidermolysis bullosa acquisita; LAD, Linear IgA bullous disease.

## Antibody transfer induced model systems

Antibody transfer-induced mouse models have been established for BP, EBA, MMP and LAD (83). The injection of pathogenic (auto)-antibodies into mice induces symptoms duplicating features of the corresponding human disease. In the case of BP, injection of rabbit IgG targeting the murine-COL17 (mCOL17) domain corresponding to the NC16A human-COL17 (hCOL17) domain, have been found to induce dermal-epidermal separation in neonatal wild-type mice as early as 1993 (87). Further development of this model led to the usage of adult mice that can reflect that BP develops in elderly patients (88, 89). Moreover, transgenic mice either carrying hCOL17 or mCOL17 expressing the human NC16A domain have been introduced and develop BP upon injection of human IgG autoantibodies (90, 91). Based on this principle, antibody transfer models have been developed for EBA and MMP (92, 93). These models show a high disease penetrance in most inbred mouse strains (94, 95).

Data from antibody transfer-induced models of EBA have been the basis to obtain orphan designation for dimethyl fumarate and coversin/nomacopan in BP (96–99). The dual complement factor 5 (C5) and leukotriene B4 (LTB4) inhibitor nomacopan was successfully evaluated in a phase 2a nonrandomized controlled trial (100, 101). More recently, again based on data in an antibody transfer model of MMP (101), a patent on the use of CXCL8 inhibitors for the treatment of MMP was filed (102). Collectively, this illustrates the applicability of antibody transfer PD mouse models for clinical translation.

## Lymphocyte transfer induced model systems

These models are based on the transfer of lymphocytes from an antigen-deficient mouse strain (with or without immunization with the respective antigen) into immunodeficient mice with the expression of the respective antigen (103). A lymphocyte transfer model has been established for BP (**Table 1**). Here, wild-type mice are immunized with human COL17 by grafting skin from hCOL17 transgenic mice. Transfer of lymphocytes from these mice into immunodeficient mice expressing the hCOL17 transgene in the skin induces a strong anti-hCOL17-specific humoral immune response. These mice develop a clinical phenotype resembling the clinical, histological, and immunopathological features of the human disease (104). This model has mainly been used to unravel the pathogenesis of autoantibody production in BP, for example, the requirement of CD4 T cells (105). Notably, IVIG treatment, a second- or third-line treatment option for BP, is also effective in this model (106). This demonstrates that the lymphocyte transfer model of BP is in principle well suited to identify and validate novel treatment strategies in BP.

## Immunization induced model systems

Immunization-induced PD model systems have been established for BP and EBA (107–109) (**Table 1**). Immunization-induced BP shows a low disease penetrance and almost exclusively manifests in female mice. Due to these limitations, this model is not widely used. By contrast, the immunization-induced EBA mouse model based on a single immunization with recombinant mCOL7 (109) induces clinical disease manifestation in 60%-80% of the mice within 4-10 weeks. Mouse strains that are susceptible to immunization-induced EBA are SJL/J and B6.SJL-H2s C3c/1CyJ mice. The latter are C57Bl6/J mice that carry the H2s haplotype from the SJL/J strain. After single immunization, peak disease severity is observed after around 10 weeks, reaching a plateau that is maintained for at least 10 months (109). Based on these characteristics, immunization-induced EBA has been used to evaluate the effects of established and emerging treatments of PD (92, 96, 108, 110, 111).

## Advantages and disadvantages of the pre-clinical animal model systems

All murine models duplicate key aspects of human PD. Furthermore, parallel use of antibody transfer-induced models and lymphocyte transfer- or immunization-induced models also allows to disentangle the afferent (loss of tolerance, autoantibody production) from the effector (autoantibody-induced tissue damage) phase. In the antibody transfer-induced models, especially when the antibodies are injected locally into the skin of the ear, clinical symptoms manifest within days following the transfer of pathogenic IgG, allowing for timely analysis. A significant limitation of these models, however, is its restriction to studying only the effector phase of the disease, as symptoms abate within days post-autoantibody transfer. Furthermore, these models are not conducive to examining the mechanisms of tolerance loss or the production of autoantibodies (83). As these models are still almost exclusively based on the transfer of rabbit or human IgG into mice, an antibody response towards rabbit or human IgG limits long-term observations (112). Conversely, lymphocyte transfer- and especially immunization-induced PD mouse models are characterized by a prolonged manifestation of a clinical PD phenotype, enabling the evaluation of long-term interventions and the investigation of factors influencing tolerance loss and autoantibody production. Yet, these models are significantly more time-consuming and resource-intensive compared to the autoantibody transfer models. There are also significant disadvantages associated with both models. Firstly, both models rely on murine signaling pathways and cell interactions, which may not accurately translate to human physiology, thus limiting their predictive value for clinical trials (83). Secondly, animal trials inherently involve animal suffering and death. Third, a significant proportion of drug development centers on specific antibodies. Often, these do not cross-react between species, which needs to be addressed and may be challenging. Hence, the potential advantages have to be carefully weighed against the burden and death of experimental animals.

## Experimental interventions in pre-clinical PD model systems for target identification and validation

These models have significantly contributed to our understanding of PD pathogenesis and have led to the identification and validation of several new therapeutic targets. Equally, these models also excluded several molecules as targets, which is at least equally important. **Table 2** summarizes the cellular and molecular targets investigated in preclinical PD mouse models. The following paragraphs highlight those targets that have emerged as most promising.

**Table 2:** Experimental interventions in pre-clinical animal models of PD.

Disease mouse model	Target	Experimental intervention	Result	Reference
Not effective				
EBA/BP	MRP8 and 14	MRP8/14 deficient mice	mice fully susceptible to disease	(219)
EBA	TREM1	TREM1 deficient mice	mice fully susceptible to disease	(220)
EBA	CCL3/MIP1α	CCL3/MIP1α deficient mice	mice fully susceptible to disease	(221)
BP	FcγRIIb	FcγRIIb deficient mice	mice fully susceptible to disease	(222)
EBA	C6	C6 deficient mice	mice fully susceptible to disease	(116)
EBA	CD130	sgp130Fc	mice fully susceptible to disease	(223)
EBA	MMP3	MMP3 deficient mice	mice fully susceptible to disease	(224)
EBA	Hsp70	anti-Hsp70-antibody	Hsp70 blockade leads to increased disease activity increased disease activity	(225)
BP	C5aR2	C5aR2 deficient mice	C5aR2 deficient mice exhibit increased disease activity	(226)
BP	FcγRII	FcγRII deficient mice	FcγRII deficient mice exhibit increased disease activity	(226)
BP	C5aR1	PMX-53	mice fully susceptible to disease	(226)
EBA	PI3Kγ	AMG319	mice fully susceptible to disease	(118)
EBA	PI3Kδ	AMG319	mice fully susceptible to disease	(118)
EBA	PI3Kα	HS-173	mice fully susceptible to disease	(118)
EBA	Caspase-1	Caspase-1 deficient mice	mice fully susceptible to disease	(227)
EBA	FcγRIIb	FcγRIIb deficient mice	FcγRIIb deficient mice exhibit increased disease activity	(228)
EBA	FcγRI	FcγRI deficient mice	mice fully susceptible to disease	(228)
EBA	FcγRII	FcγRII deficient mice	mice fully susceptible to disease	(228)
EBA	IL6	anti-IL6 antibody	IL6 blockade leads to an increase in disease severity	(223)
EBA	IL6	IL6 deficient mice	IL6 deficient mice exhibit increased disease activity	(223)
EBA	Mannan binding lectin	MLB deficient mice	mice fully susceptible to disease	(229)
EBA	GATA-1	ΔdblGATA mice	mice in which eosinophils are absent are fully susceptible to disease	(115)
EBA	multiple	niclosamide	mice fully susceptible to disease	(123)
EBA	thioredoxin reductase	auranofin	mice fully susceptible to disease	(123)
EBA	β1 adrenoceptor	dobutamine	mice fully susceptible to disease	(123)
EBA	multiple	dipyridamole	mice fully susceptible to disease	(123)
EBA	tubulin	colchinine	mice fully susceptible to disease	(123)
EBA	CD11b	CD11b deficient mice	CD11b deficient mice exhibit increased disease severity	(230)
Little effectiveness				
EBA	PI3Kδ	LAS191954	small reduction of disease activity	(92)
EBA/BP/MMP	PI3Kδ	Parsaclisib	small reduction of disease activity	(101)
EBA	PDE4	roflumilast	roflumilast reduces disease activity *in vivo*	(111)
EBA	PDE4	rolipram	rolipram shows inhibitory capabilities	(111)
EBA/BP	TNF	TNF receptor fusion protein etanercept	eternacept treatment leads to small reduction of disease activity	(231)
EBA/BP	TNF	anti-TNF antibody	TNF blockade leads to more reduction of disease activity compared to eternacept	(231)
BP	FcγR	FcγR deficient mice	FcγR deficient mice exhibit reduced disease activity	(206)
BP	FcRn	IgG2c-ABDEG	FcRn blockade via IgG2c-ABDEG slightly reduces disease activity	(206)
BP	C5aR1	C5aR1 deficient mice	C5aR1 deficient mice exhibit reduced disease activity	(206)
BP	C5aR1	C5aR1 deficient mice	C5aR1 deficient mice exhibit slightly reduced disease activity	(226)
EBA	Flii	altering Flii levels in mice	Overexpression of Flii leads to increased disease severity while redution has beneficial effects	(232)
EBA	Flii	anti-Flii antibodies (FnAbs)	topical application of FnAbs leads to decreased disease severity by ~20%	(233)
EBA	PI3Kδ	IC87114	PI3Kδ blockade via IC87114 slightly reduces disease activity	(118)
EBA	PI3Kδ	GDC-0941	PI3Kδ blockade via GDC-0941 slightly reduces disease activity	(118)
EBA	C1q	C1q deficient mice	Only at the end of observation C1q deficient mice show slightly reduced disease activity	(229)
Medium effectiveness				
EBA	IFNγ	anti-IFNγ-antibody	IFNγ blockade reduces disease activity (~50% disease reduction at peak disease and highest Ab concentration)	(234)
EBA	FcγR	sCD32	FcγR inhibition with sCD32 leads to reducted disease severity but shows more inhibitory effects *in vitro*	(235)
BP/EBA	multiple	Methylpredsinolone	Methylprednisolone treatment reduces disease activity by ~50%	(236)
BP/EBA	multiple	SB203580	SB203580 treatment reduces disease activity by ~50%	(236)
BP/EBA	multiple	U0126	U0126 treatment reduces disease activity by ~50%	(236)
EBA	Granzyme B	GzmB deficient mice	affected ear and body area is reduced by 55% and 45% respectively	(237)
EBA/BP	Granzyme B	VTI-1002	topical VTI-1002 treatment reduces disease activity by ~50% in a local EBA and BP model	(237)
BP	C5	C5 deficient mice	C5 deficient mice exhibit ~50% reduced disease activity	(226)
EBA	C5aR2	C5aR2 deficient mice	C5aR2 deficient mice exhibit reduced disease activity	(238)
EBA	C5aR2	C5aR2 deficient mice	C5aR2 deficient mice exhibit reduced disease activity	(239)
EBA	multiple	dyclonine hydrochloride	preventive topical administration of dyclonine hydrochloride ameliorates disease severity	(240)
EBA	PI3Kα	alpelisib	PI3Kα blockade via alpelisib reduces disease activity	(118)
EBA	PI3Kγ	AS604850	PI3Kγ blockade via AS604850 reduces disease activity	(118)
EBA	multiple	Propanolol	Propanolol treated mice exhibit reduction of disease activity when applied topically and systemically	(241)
EBA	CXCR1/2	DF2156A	CXCR1/2 blockade via DF2156A reduces disease activity in passive transfer model and immunization model	(242)
EBA	CXCR1/2	CXCR2-deficient mice	CXCR2-deficient mice exhibit reduced disease activity similar to pharmacological inhibition	(242)
EBA	C5/LBT4	Coversin	Coversin treated mice exhibit reduced disease activity	(97)
EBA	C5/LBT4	L-Coversin	L-Coversin treated mice exhibit reduced disease activity, although therapeutic effect of Coversin is stronger	(97)
EBA	IL1R	IL1R deficient mice	IL1R deficient mice ehibit reduced disease activity	(227)
EBA	IL1b	anti-IL1b antibody	IL1b blockade reduces disease activity	(227)
EBA	IL1	anakinra (IL1ra)	therapeutically applied anakinra managed to reduce disease activity by ~30%	(227)
EBA	multiple	DMF	DMF treated mice exhibit reduction of disease severity by 50-60% when applied prophylactically and therapeutically	(96)
EBA	factor B	fB deficient mice	fB deficient mice exhibit reduced disease severity by ~55%	(229)
EBA	CARD9	CARD9 deficient mice	CARD9 deficient mice exhibit reduction of disease severity by ~60%	(243)
EBA	5-lipoxygenase	zileuton	5-LO blockade via zileuton reduces disease activity by ~50%	(115)
EBA	multiple	amodiaquine	amodiaquine treatment leads to reduced disease activity	(123)
EBA	dopamine receptor 2	apomorphine	D2R blockade via apomorphine leads to reduced disease activity	(123)
High effectiveness				
EBA	FcγRIV	anti-FcγRIV antibody	FcγRIV blockade blocks disease in 80% of mice	(228)
EBA	FcγRIV	FcγRIV deficient mice	FcγRIV deficient mice are disease resistant	(228)
EBA	γ-chain of activating FcγR	γ-chain deficient mice	γ-chain deficient mice are disease resistant	(228)
EBA	FcγRIV	anti-FcγRIV antibody	FcγRIV blockade fully blocks disease development	(244)
EBA	SYK	BAY61-3606	pharmacological SYK blockade prevents disease induction *in vitro* and *in vivo*	(245)
EBA	SYK	PRT062607	PRT062607 shows similar *in vitro* results as BAY61-3606	(245)
EBA	SYK	entospletinib	entospletinib reduces disease activity *in vitro* with human samples	(246)
EBA	SYK	lanraplenib	lanraplenib reduces disease activity *in vitro* with human samples	(246)
EBA	SYK	mice with hematopoietic-specific SYK deficiency	mice with hematopoietic-specific SYK deficiency are disease resistant	(246, 247)
EBA	Neutrophil cytosolic factor 1	Nfc1 deficient mice	Ncf1 deficient mice are disease resistant in 95% of cases	(149)
EBA/BP	NADPH oxidase	Diphenylene iodonium	Diphenylene iodonium abolishes dermal-epidermal separation *ex vivo*	(149)
EBA	Granulocytes	anti-Gr-1 antibody	anti-Gr-1 antibody treated mice are disease resistant until day 6 of treatment	(149)
EBA	CD18	CD18 deficient mice	CD18 deficient mice are disease resistant	(149)
EBA	IgG glycosylation	EndoS	EndoS-pretreatment of otherwise pathogenic IgG failed to induce clinical disease	(248)
EBA	C5aR1	C5aR1 deficient mice	C5aR1 deficient mice exhibit delayed disease onset and reduces disease activity	(116)
EBA	C5aR1/2	A8Δ71–73	C5aR1/2 blockade via A8Δ71–73 reduces disease activity	(116)
EBA	factor B	anti-fB antibody	fB blockade leads to reduced disease activity by ~90% in fully treated mice and ~60% in mice treated from day 5	(116)
EBA	C5	anti-C5 antibody	C5 blockade reduces disease activity	(116)
EBA	Hsp90	17-DMAG	Hsp90 blockade via 17-DMAG reduces disease activity by ~90% in prophylactic and therapeutic treatment	(249)
EBA	Hsp90	TCBL-145	Hsp90 blockade via TCBL-145 reduces disease activity by ~90% in prophylactic and therapeutic treatment	(249)
EBA	IL6	recombinat IL6	mice injected with recombinant IL6 are almost protected from disease	(223)
EBA	IL1	anakinra (IL1ra)	anakinra reduces disease severity by ~75%	(223)
EBA	multiple	Calcitriol	systemic calcitriol treatment leads to reduction of disease activity in several models as well as *in vitro*	(250)
EBA	PLCγ2	PLCγ2 deficient mice	PLCγ2 deficient mice are disease resistant	(251)
EBA/BP	RORα	RORα deficient mice	RORα deficient mice are disease resistant	(95)
EBA/BP	RORα	RORα heterozygous mice	RORα heterozygous mice show reduced disease severity	(95)
EBA/BP	RORα	SR3335	pharmacological RORα blockade reduced disease severity *in vivo* and *in vitro*	(95)
EBA	GM-CSF	GM-CSF deficient mice	GM-CSF deficient mice exhibit strong reduction of disease activity	(252)
EBA	GM-CSF	anti-GM-CSF antibody	anti-GM-CSF antibody treated mice exhibit a reduction of disease activity similar to genetic intervention	(252)
EBA	C5aR	C5aR deficient mice	C5aR deficient mice exhibit reduced disease activity by ~90%	(114)
BP	IL17A	IL17A deficient mice	IL17A deficient mice are disease resistant	(253)
BP	IL17A	anti-IL17A antibody	anti-IL17A antibody treated mice exhibit reduced disease activity	(253)
BP	MMP9	MMP9 deficient mice	MMP9 deficient mice are disease resistant	(224)
BP	Plg	Plg deficient mice	Plg deficient are were disease resistant	(224)
EBA	Src kinases	Src kinases deficient mice	Src kinases deficient mice are disease resistant	(254)
EBA	FcRn	FcRn deficient mice	disease onset delay and strong reduction of disease activity, however higher Ab doses override protective effect	(255)
BP	FcRn	FcRn deficient mice	FcRn deficient mice are disease resistant	(222)
BP	FcRn	high-dose human IgG	high-dose human IgG treated mice exhibit reduced disease activity	(222)
BP	Gelatinase B	Gelatinase B deficient mice	Gelatinase B deficient mice are disease resistant	(256)
BP	FceR	FceR deficient mice	FceR deficient mice are disease resistant	(226)
BP	FcγRIV	FcγRIV deficient mice	Fcγr4 deficient mice exhibit reduced disease activity by 75%	(226)
EBA	PI3Kβ	TGX-221	PI3Kβ blockade via TGX-211 almost abolished disease activity in systemic treatment and reduces disease activity in topical treatment	(118)
EBA	PI3Kβ	PI3Kβ deficient mice	PI3Kβ-deficient mice exhibit reduced disease activity, while βKO bone marrow chimeras are even more protected	(117)
EBA	5-lipoxygenase	5-LO deficient mice	5-LO deficient mice are disease resistant	(115)
EBA	BLT1	BLT1 deficient mice	BLT1 deficient mice are disease resistant	(115)
EBA	Ly6G	anti-Ly6G antibody	Ly6G blockade and resulting neutrophil depletion leads to a strongly reduced disease activity until day 10	(115)
EBA	tubulin	docetaxel	tubulin blockade via docetaxel reduces disease activity during prophylactic treatment	(123)
EBA	ribonucleotide reductase	gemcitabine	RNR blockade via gamcitabine reduces disease activity during prophylactic treatment	(123)
EBA	succinate dehydrogenase	pyrvinium pamoate	SDH blockade via pyrvinium pamoate reduces disease activity during prophylactic treatment and halted disease progression in therapeutic treatment	(123)
EBA	Selective estrogen receptor modulator (SERM)	tamoxifen	SERM blockade via taximofen reduces disease activity	(123)

BP, Bullous pemphigoid; MMP, Mucous membrane pemphigoid; EBA, Epidermolysis bullosa acquisita.

Dimethyl fumarate (DMF) is approved for psoriasis (Skilarence^®^) and multiple sclerosis (Tecfidera^®^). In the antibody transfer-induced EBA mouse model, DMF reduced EBA severity by more than 50%, and, even more strikingly, led to resolving of lesions when used in therapeutic settings in the immunization-induced EBA mouse model. Investigating of the mode of action (MOA) demonstrated that DMF impairs neutrophil activation *in vitro* as well as tissue disruption *ex vivo* in an ERK1/2, p38 MAPK and Akt-dependent manner (96). Based on these results, orphan designation (98) was granted by the European Medicines Agency (EMA). Following this publication, a BP patient was successfully treated with DMF (113). The planned phase 2 clinical trial aiming to evaluate the safety and efficacy of DMF in BP could, however, not be initiated. Nomacopan, also known as Coversin, received orphan designation for the treatment of BP, again based on data from antibody transfer-induced EBA (97, 99). As a dual inhibitor of leukotriene B4 (LTB4) and complement component C5, its efficacy was validated in the antibody transfer-induced EBA model, following the identification of LTB4 and the C5a/C5aR1-axis as key factors for autoantibody-induced tissue damage in the model (97, 114–116). The subsequent phase 2a clinical trial including 9 BP patients showed that nomacopan is safe and may have therapeutic benefits for suppressing acute disease flares (100). The ß-isoform of phosphoinositide 3-kinase (PI3Kß) was identified as a target in a study that showed that genetic and pharmacological inhibition of PI3Kß leads to substantial disease protection in antibody transfer-induced EBA. Regarding the MOA, PI3Kß mediates several immune complex (IC)-elicited neutrophil responses *in vitro*, including release of reactive oxygen species (ROS) (117). A follow-up study, investigating the impact of PI3K-inhibitors with different selectivity for PI3Kα, β, γ or δ, supported these findings. Here, only the Pi3Kβ-selective TGX-221 impaired the clinical disease manifestation of antibody transfer-induced EBA – of note, also when topically applied. In parallel, the impact of different PI3K-inhibtors on neutrophil functions was evaluated. In these experiments, TGX-221 impaired IL-8-induced neutrophil migration, spreading of neutrophils on immobilized IC and IC-induced ROS release from neutrophils (118). Given that PI3Kβ-selective inhibitors are in clinical trials for other indications (119, 120), targeting this pathway (preferably by topical application) in PD seems valid.

## Alternatives to pre-clinical model systems in target identification and validation

**Figure 2:**
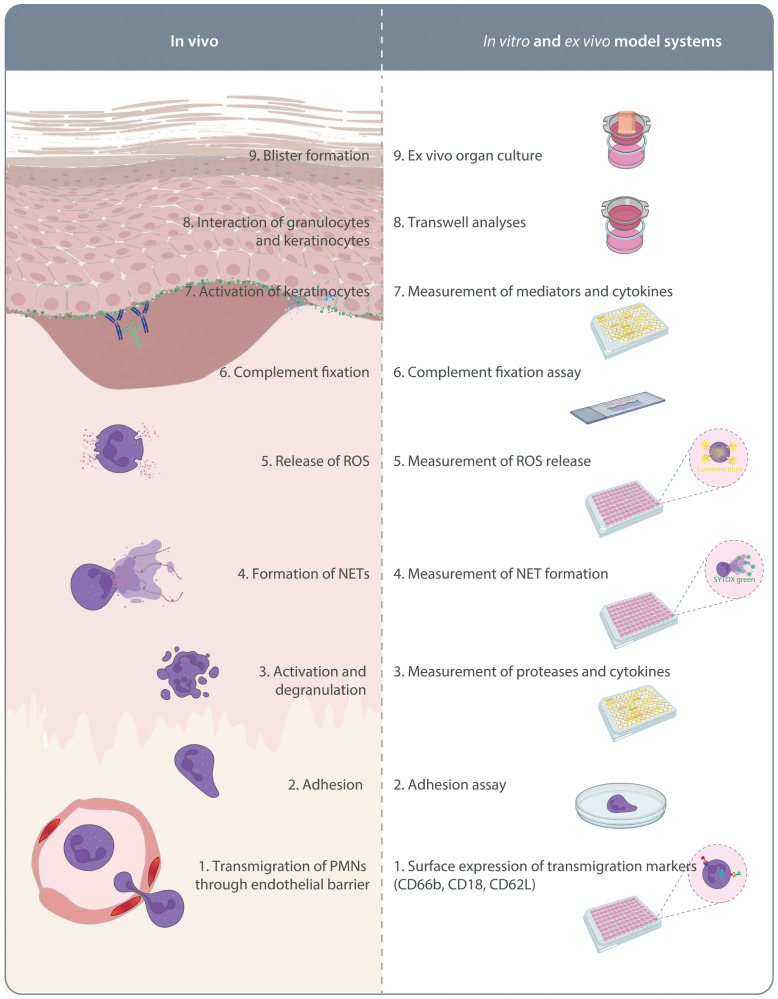
*In vitro* and *ex vivo* model systems of pemphigoid diseases. On the left side the pathogenesis of autoantibody-mediated tissue pathology in pemphigoid diseases is indicated. This is initiated by migration of polymorphonuclear leukocytes (PMN) into the skin. On the right side, assays mimicking these aspects of pemphigoid disease pathogenesis are indicated. These assays are described in detail in the text.

As detailed above, investigations using pre-clinical model systems of PD additionally employed *in vitro* methods to identify the MOA of the tested compounds. Although not systematically addressed, a large proportion of the compounds shown to inhibit key pathogenic pathways in PD (**Figure 1**) were also able to impair the onset of antibody transfer-induced PD or have therapeutic effects in the immunization-induced models. There is a clear political commitment in the European Union to accelerate phasing out of animal testing (121), and the FDA modernization act “authorizes the use of certain alternatives to animal testing, including cell-based assays and computer models, to obtain an exemption from the Food and Drug Administration to investigate the safety and effectiveness of a drug” (https://www.congress.gov/bill/117th-congress/senate-bill/5002). To address this and to strictly implement the 3R principle (refine, reduce, replace) (122), we trust that routine and innovative new human-based *in vitro* or *ex vivo* models allowing testing of potential compounds will - to a certain extend - lead to prediction of efficacious therapeutics. These will not only replicate the outcome of *in vivo* experiments and thus significantly reduce the need for animal experimentation (**Figure 2**), but also provide assays in which human-targeting drug can be evaluated. This principle has recently partially been implemented in a large-scale screening endeavor to repurpose drugs for modulation of innate and acquired immune responses (123). In this paragraph, we thus will discuss alternative strategies to replace rodent-utilizing, preclinical model systems to align with the 3R principles (124). The below proposed *in vitro, ex vivo and ex vivo* systems align with the current understanding of PD pathogenesis outlined in **Figure 1**.

## PD autoantigen-specific T cells

### Anti-CD3/CD28-induced T cell activation

Most investigations used non-antigen-specific T cell stimulations using anti-CD3 and anti-CD28 antibodies (123). Results from these investigations can potentially predict the impact on autoreactive T cells. However, as pan-T cell targeting treatments are expected to be associated with considerable adverse events (125), this, relative basic method may be used to screen larger libraries to select compounds for further *in vitro* testing.

### Detection of antigen-specific T cells

Flow cytometry can be used to detect antigen-specific T cells in PD (126, 127). One can envision combining this with specific assays targeting these antigen-specific T cells (128). Yet, these assays need to be established for BP.

### Antigen-reactive T cell enrichment

The ARTE technology allows investigating antigen-specific T cell responses and has recently been used to in-depth characterize autoantigen-specific CD4 T cells in several autoimmune diseases, including BP (126). Whilst ARTE primarily aims to decipher the phenotype of antigen specific T cells from human samples, it could also be used to characterize these cells following an experimental intervention *in vitro*. The latter, to be developed, assays would be a potential asset to predict the efficacy of these intervention in pre-clinical model systems.

## PD autoantigen-specific B cells

### IL-21 and anti-CD40-induced B cell activation

B cells can be stimulated *in vitro* by IL-21 and anti-CD40. This unspecific B cell stimulation has been used to identify B cell inhibitory compounds in a drug screening attempt. Here 1,200 compounds were screened for their potential B cell inhibitory activity. Screening and *in vitro* validation identified five drugs that were subsequently tested in pre-clinical PD mouse models. Three of the five compounds were indeed able to impair the induction of immunization-induced EBA (123). This highlights that this assay is indeed able to identify B cell modulatory compounds that are effective *in vivo*. These compounds are likely to non-specifically suppress B cell activation, which could lead to potential adverse events, thereby limiting their clinical applicability.

### Detection of antigen-specific B cells

Similar considerations, as outlined above for T cells, apply. Immunophenotyping of PD patients’ B cell populations has been described (129), and these methods may be modified to detect changes upon treatment. In addition, ELISpot may be used to determine B cell functions following an *in vitro* manipulation (130).

## Neonatal Fc receptor

### Maintaining high levels of IgG autoantibodies

One of the main functions of the neonatal Fc receptor (FcRn) is to maintain high levels of circulating IgG concentrations in the blood. This is achieved by protecting IgG from lysosomal degradation after uptake by endothelial cells (131, 132). Inhibition of the FcRn lowers circulating IgG concentrations, including those of IgG autoantibodies, and this has been demonstrated to be effective in autoantibody-mediated diseases (133). Assays to determine the impact of compounds targeting the FcRn have been developed (134), but so far, have not been used in PD research. These cellular recycling assays would be, however, valuable to determine if compounds to be tested in pre-clinical PD model systems have an impact on IgG turnover.

## PD autoantibody binding to target cells

### Mediator release from keratinocytes

The current understanding of pemphigoid disease pathogenesis considers keratinocytes to be key effector cells in mediating autoantibody-induced tissue pathology (135). This understanding is based on the discovery of IL-6 and IL-8 release from keratinocytes incubated with anti-BP180 antibodies (136). The principle of this assay is still in use today. However, systematic investigations on the impact of drugs on autoantibody-induced mediator release and their subsequent effects in keratinocyte model systems is so far lacking.

### Internalization of autoantigens

Another direct impact of PD autoantibodies on keratinocytes is the internalization of autoantigens (137). This weakening of the adhesion of hemidesmosomes to the lamina densa, followed by the inflammatory events triggered by autoantibody binding could be the reason for the ultrastructural site of the split formation. Like with many of the *in vitro* model systems in PD, a systematic approach aiming to correlate *in vitro* observations to efficacy in pre-clinical models is lacking.

## Complement-fixation and activation through tissue-bound immune complexes

Initially developed as a diagnostic assay for PG (138), modifications of the complement (C)-fixation assay can be used to model complement activation in PD. For this, cryosections of human skin are incubated with PD antibodies. After washing, a complement source is added. Endpoints include determination of C3 deposits at the dermal-epidermal junction and evaluation of complement cleavage products in the supernatant (110, 139). So far, no insight on the prediction of outcomes regarding *in vivo* model systems has been obtained. However, compounds positively evaluated in the complement fixation assay showed efficacy in phase 1 clinical trials including BP patients (140), or other pre-clinical disease models (141).

## Immune complex-induced neutrophil activation

### Neutrophil spreading on fixed IC

Spreading is one of the first events after binding of neutrophils to IC. The assay duplicating this key event has been adopted for PD in 2010 (142). This assay has been used in several publications relating to PD. Yet, the prognostic value of this test for predicting efficacy in pre-clinical model systems has not been systematically evaluated.

### IC-induced changes in neutrophil surface molecule expression

Changes in expression of adhesion molecules is another hallmark of IC-induced neutrophil activation, indicating altered migratory capabilities (e.g., CD18, CD62L) or degranulation (e.g., CD66) (143). Regarding PD, flow cytometry has been used to address IC-induced changes in neutrophils (111). Due to the relatively limited number of publications utilizing this method, the predictive value of altered surface molecule expression on IC-activated neutrophils regarding effects in pre-clinical PD model systems remains to be elucidated.

### IC-induced mediator release from neutrophils

Mediator release is another hallmark of neutrophil activation (143). In PD, lipid mediators, cytokines and complement are key soluble mediators (18). So far, the cellular source of these mediators remains to be determined. Given the central role of soluble mediators in neutrophil activity regulation and resolving inflammation as well as the relative straight-forward and highly up-scalable methods for determining these soluble mediators (143–145), analysis of these for prediction of treatment outcomes holds a high potential.

### IC-induced ROS release from neutrophils

The “ROS-release assay” is widely used to investigate IC-induced neutrophil activation in PD (146). In this assay, immune complexes are generated in 96-well plates which are incubated with freshly isolated human or (more rarely) mouse neutrophils. These, in an Fc-gamma, or Fc-alpha-receptor mediated fashion, become activated (147, 148) and release ROS, which are detected by chemiluminescence. The “ROS-release assay” has been the basis to identify drug candidates to inhibit IC-induced neutrophil activation in a repurposing study. This *in vitro* screening identified six from a total of 1,200 compounds. All six were then evaluated for their safety and efficacy in the antibody transfer-induced EBA mouse model, where 3 of 6 compounds impaired induction of experimental EBA (123). Overall, this indicates that the “ROS-release assay” can be used to drastically reduce the requirement for animal experimentation. However, half of the compounds identified by the “ROS-release assay” as potential new treatment options of PD, failed validation in pre-clinical animal models.

### IC-induced, neutrophil-mediated dermal-epidermal separation *ex vivo* (cryosection assay)

The cryosection assay duplicates the subepidermal blistering observed in PD. Here, cryosections of normal human (and rarely mouse) skin are incubated with PD autoantibodies. After washing, neutrophils from healthy donors are added. Again, these are activated by binding of Fc-gamma, or Fc-alpha-receptor to the tissue-bound IC. Ultimately, this leads to neutrophil spreading (see above), ROS- and protease-release, which mediate the dermal-epidermal separation (149, 150). The cryosection assay is not applicable to high throughput and is thus mainly used to validate findings from the ROS-release assay. With regard to implementation of the 3R principles, the cryosection assay may be used following the ROS-release assay to further limit the number of compounds to be used *in vivo*.

### 3D human skin models

A recent paper described a 3D human skin equivalent that was incubated with BP180-affinity-purified IgG from BP patients. This polyclonal anti-BP180 induced BP180 internalization and led to subepidermal split formation (151). Of note, addition of an FcRn inhibitor reduced IgG deposition along the basement membrane of the keratinocytes, indicating that this assay may also be used to test for FcRn function. We believe that this model holds the potential to investigate autoantibody-induced tissue damage in pemphigoid diseases in great depth. Modifications, for example, addition of leukocytes would allow to investigate the interplay between keratinocytes, autoantibodies and leukocytes *in vitro*. Use of skin biopsies from healthy donors or pemphigoid disease patients would allow to additionally investigate the impact of resident immune cells within the skin. Especially these *ex vivo* models could significantly reduce the need for animal testing when investigating autoantibody-induced tissue damage.

## Other PD-related neutrophil functions

### Neutrophil migration towards IL-8 or C5a

IL-8 (and its’ mouse homologues) and C5a are central in PD pathogenesis (36, 114, 116). In PD, IL-8- or C5a-induced neutrophil migration has been used in several studies (95, 96). In some, but not all of the studies, the migratory ability of neutrophils *in vitro* correlated well with those observed in pre-clinical model systems. Thus, the use of these assays to reduce or replace animal experimentation needs to be elucidated.

In summary, a systematic evaluation of the above-described *in vitro* models is warranted to determine whether one, or more likely a combination of several *in vitro* systems, can effectively predict *in vivo* effects of molecules or drugs. This approach is expected to significantly reduce the need for animal experimentation. Thus, a systematic investigation to address the predictive value of PD *in vitro* assays for compound efficacy in pre-clinical models is urgently needed.

## Use of real-world-data

Advances in the availability and analysis of real-world data (RWD) have expanded the possibilities and applicability to generate evidence and thereby allowing regulatory agencies, such as the FDA, to base decisions on RWD. Thus, the FDA is putting more emphasis on RWD to enhance therapeutic drug development and strengthen regulatory oversight throughout the medical product lifecycle (152). The data quantity and quality of some RWD databases allows to predict disease onset and allows to stimulate clinical trials (153). In the context of PD, a recent study compared the risk of death and relapse in BP patients treated either with topical or systemic corticosteroids. Here, risk of death was increased in patients with BP exposed to any dose of systemic corticosteroids versus BP patients treated with topical clobetasol propionate (22). This study can in principle be adopted to any other drug. As a limitation, however, insights into efficacy are limited because disease severity scores are not available and drug dosages are not recorded.

## Drug repurposing

Drugs approved for other indications may be repurposed for PD. An excellent example for this within the field of pemphigus and pemphigoid is the use and licensing of rituximab in pemphigus. The CD-20 antibody rituximab was initially licensed for the treatment of patients with relapsed or refractory B cell non-Hodgkin’s lymphoma in 1997 (154). Due to the drug’s MOA, specifically B cell depletion, it was given to a 30-year-old woman with refractory pemphigus vulgaris, resulting in partial remission (155). Following two randomized controlled clinical trials, rituximab in combination with prednisolone is now the standard of care in pemphigus (156, 157). For PD, controlled clinical trials of the safety and efficacy of rituximab are so far lacking. However, data from retrospective analyses indicate a moderate effect of rituximab in PD (158–160).

In PD, three drugs, in addition to DMF which is discussed above, licensed for other indications seem to be effective: The anti-IL4Rα antibody dupilumab, the anti-IgE antibody omalizumab and Janus kinase (JAK) inhibitors (JAKi). Of these, dupilumab holds the largest promise. In 2017, dupilumab was licensed for atopic dermatitis (161, 162). Based on the Th2-phenotype of T cells in BP (163), and the intense pruritus in BP, dupilumab was successfully used off-label in several BP patients by different medical practitioners (164, 165). Recently, a multicenter, ambispective cohort study investigated the safety and efficacy of dupilumab in 103 BP patients. Overall, dupilumab was safe and effective: Adverse events were observed in 13 of 103 patients and were mostly mild. Complete remission was achieved in 53.4% of BP patients within 4 weeks and 95.7% by week 52 (166). A recent press release from Sanofi on their randomized, phase 2/3, double-blind, placebo-controlled study evaluating dupilumab in BP reported that the study met all primary endpoints. Specifically at week 36 of the study, 20% of BP patients in the treatment arm experienced sustained disease remission, compared to 4% in the control arm (167). Similar findings were made in a large retrospective cohort study from China (168). Based on these considerations, approval for dupilumab in BP is expected within the next 1-2 years. Of note, a rapid and sustained response to the IL-13 targeting antibody tralokinumab has been reported in one patient with BP (169), that replicated in a larger case-series (170). At this point, this data does not allow to draw any final conclusions. However, the precise contribution of IL-4 and IL-13 to PD pathogenesis remains to be fully elucidated.

The anti-IgE antibody omalizumab was first licensed in 2003 for the treatment of asthma (171). Subsequently, it was also licensed for other type 2 inflammatory diseases including chronic spontaneous urticaria, chronic rhinosinusitis with nasal polyps and most recently also for food allergies (172–174). There is a body of evidence pointing towards a potential contribution of IgE in PD, especially BP: Elevated levels of total IgE are observed in BP. In some patients, antigen-specific IgE can be detected. Last but not least, in an antibody-transfer model, antigen-specific IgE antibodies elicited experimental BP when transferred into mice (175, 176). In 2009, a BP patient was successfully treated with off-label use of omalizumab (177). These results were confirmed in several case reports. More recently, a multicenter retrospective study conducted by the French Study Group on Autoimmune Bullous Diseases investigated the effectiveness and safety of omalizumab in BP patients. The study included 100 BP patients. Omalizumab led to complete remission in close to 80% of the patients and displayed a favorable safety profile. Of note, complete remission was more frequently observed in patients with an increased serum baseline level of antigen-specific IgE autoantibodies targeting BP180 (178). Taken together, omalizumab is another promising candidate for the treatment of BP. This may also be applicable to other pemphigoid diseases, e.g., MMP (179), but data on IgE and/or omalizumab is rather scant for PD other than BP.

More recently, JAKi have been licensed for several non-communicable inflammatory diseases, including their topical administration in atopic dermatitis and vitiligo (180, 181). Given the boxed warning of JAKi concerning their cardiovascular risk profile (182), topical application in PD, especially BP, would be the preferred application route. The recent case reports on the off-label use of JAKi in PD is mostly based on their increased expression at the site of inflammation (183–185). So far, six patients with BP (186–190), four patients with MMP (191, 192), and one patient with LPP (193) have been reported to have responded to off-label JAKi treatment. More data on the efficacy, and most notably on the safety of JAKi in PD are warranted. However, based on the evidence available so far, topical JAKi may be useful in BP, whilst their systemic application may best be applicable for treatment refractory MMP or EBA.

Please note that most of these studies originated from Europe or the US, and that ethnicity is seldom indicated. As we have recently demonstrated, consideration of racial disparities in autoimmune skin blistering diseases is, however, quite important (194).

## Treatment perspectives

### Biologics: dupilumab and omalizumab

As seen in other dermatological disorders (161, 162), biologics, specifically dupilumab and omalizumab, are most likely to change the treatment landscape of PD significantly. Although current available treatments, i.e. dupilumab and omalizumab, seem to be less efficient compared the gold standard corticosteroid treatment, their main advantage is the far more favorable adverse event profile. Thus, corticosteroids will be potentially used to induce rapid remission, whilst remission is maintained by long-term treatment with either dupilumab or omalizumab.

### Methotrexate

Methotrexate (MTX) has long been used as an adjuvant treatment for BP (195). A randomized trial from the French Study Group on Autoimmune Bullous Diseases compared the efficacy and safety of topical corticosteroid therapy with or without low dose MTX, where the topical corticosteroid treatment was stopped after 4-6 weeks in the MTX arm, and was continued in the control arm for nine months. A total of 300 patients were screened, but only a small fraction of BP patients was recruited due to MTX-related exclusion criteria. The remission rates were 75% in the MTX arm and 57% in the topical corticosteroid arm, which reached statistical significance. The number of severe adverse events and mortality rate was similar between the groups (196). Taken together, this indicates that MTX is a good alternative to continued (topical) corticosteroid treatment, that is, however, only applicable to 10-15% of BP patients.

## How to facilitate translation

In conclusion, the number of potential targets and molecules identified (**Table 2**) significantly exceeds the compounds that have been evaluated to date and hold promise for future PD treatment. In addition, those emerging PD treatments are so far exclusively limited to BP management. For all other PD, the pipeline for new treatments is practically non-existent. Arguably, some of the compounds effective in BP can potentially be repurposed for other PD. In the authors’ opinion, overcoming this challenge requires a joint effort from industry and academia, supported by robust model systems, including also *in vitro* and *ex vivo* human models, meticulous clinical observations, and a shared commitment to improving patient well-being through translational research.
